# Biomechanical effects of osteoporosis on adjacent segments after posterior lumbar interbody fusion: A finite element study

**DOI:** 10.12669/pjms.37.2.3223

**Published:** 2021

**Authors:** Chenchen Zhang, Minmin Chang, Renwen Zhang, Shujie Tang

**Affiliations:** 1 Chenchen Zhang, School of Chinese Medicine, Jinan University, Guangzhou, 510630, China; 2 Minmin Chang, School of Chinese Medicine, Jinan University, Guangzhou, 510630, China; 3 Renwen Zhang, School of Chinese Medicine, Jinan University, Guangzhou, 510630, China; 4 Shujie Tang, School of Chinese Medicine, Jinan University, Guangzhou, 510630, China

**Keywords:** Adjacent segmental degeneration (ASD), Finite element study, Osteoporosis, Posterior lumbar interbody fusion (PLIF)

## Abstract

**Objective:**

To investigate the biomechanical effects of osteoporosis on adjacent segments after posterior lumbar interbody fusion (PLIF).

**Methods:**

This study was designed and conducted in the Traumatology and Orthopedics Laboratory, School of Chinese Medicine, Jinan University, Guangzhou, China, between December 2019 and February 2020. A healthy finite element model of L3-S1 was developed along with one PLIF model and one PLIF with osteoporosis model. Based on a hybrid test method, the inferior surface of S1 was entirely fixed, and a preload of 400N combined with an adjusted moment was imposed on the superior surface of L3 in each model to simulate flexion, extension, lateral bending and axial rotation. The intradiscal pressure (IDP), shear stress on annulus fibrosus, and the range of motion (ROM) of L3-L4 and L5-S1 were calculated and compared.

**Results:**

In each direction, the highest value of IDP and shear stress on annulus fibrosus at L3-L4 and L5-S1 was found in the PLIF model, and the lowest value in the healthy model. The largest ROM at L4-L5 appeared in the healthy model, and the smallest value in the PLIF model in each direction. At L3-L4 and L5-S1, the highest ROM in most directions was found in the PLIF model, followed by the PLIF with osteoporosis model, and the lowest value in the healthy model.

**Conclusions:**

Osteoporosis can decrease IDP, shear stress on annulus fibrosus, and ROM at adjacent levels, and slow down the development of ASD after PLIF.

## INTRODUCTION

Adjacent segmental degeneration (ASD) is a common complication after lumbar fusion. It was reported that the incidence of radiographic ASD ranged from 8 to 100%, and symptomatic ASD ranged from 5.2 to 18.5%.^1^ Many factors, such as normal aging process, increased body mass index, and female gender, have been reported to have influence on ASD, but biomechanical factors play a key role in its pathogenesis.^2^ As a result of increased rigidity, lumbar fusion results in hypermobility and stress concentration at adjacent levels, and subsequently causes the occurrence of ASD.^2^,^3^

As a skeletal-metabolic disease, osteoporosis affects almost 200 million cases in the world.^4^ Characterized by deterioration of bone tissue and low bone mass density, the disease is prevalent in postmenopausal and aged population.^5^ With age, the patients who receive fusion surgery may suffer from osteoporosis. Based on animal experiments, Zhou suggested that osteoporosis could aggravate the development of ASD.^5^ In a systematic review, Hashimoto advocated that osteoporosis was one of the risk factors for ASD.^6^ However, in clinical studies, Bagheri found no significant difference in the incidence of osteoporosis between ASD and non-ASD groups,^2^ and Min and colleagues also concluded the same conclusion.^7^ So far, the viewpoints are still controversial.

In our opinion, when osteoporosis occurs, decreased bone mineral density can reduce the stiffness, influence the stress distribution, and lead to significant changes of the biomechanical features in the lumbar spine,^8^ so osteoporosis may affect the initiation and progression of ASD. In addition, compared with animal experiments and clinical studies, finite element technique presents many advantages in evaluating biomechanical changes in the lumbar spine after spinal surgeries, facilitating a comparative study between models with different loading conditions.^9^ As a result, we speculate that the correlation between osteoporosis and ASD can be clarified using finite element studies. However, up to now, no finite element studies have been performed on this issue.

Posterior lumbar interbody fusion (PLIF) is one of the commonly performed fusion modes in the treatment of lumbar spinal disorders, and a large number of ASD cases have been reported after PLIF.^10^ Therefore, in this study we established one healthy model, one PLIF model, and one PLIF with osteoporosis model, to investigate the biomechanical effects of osteoporosis on ASD.

## METHODS

**Fig.1:**
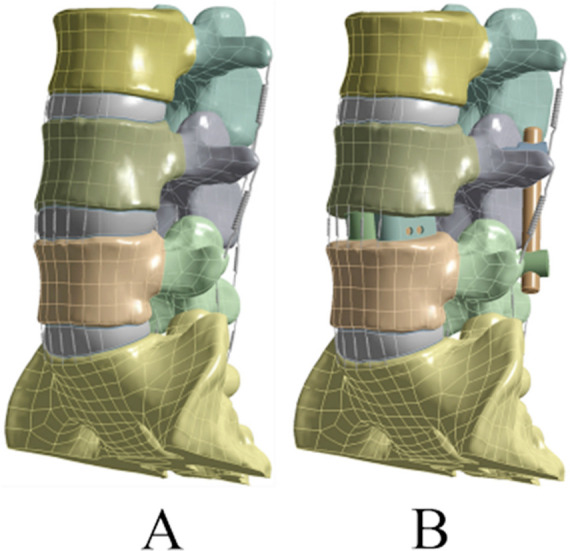
The finite element models in the current study (A: healthy model, B: posterior lumbar interbody fusion model).

This study was designed and conducted in the Traumatology and Orthopedics Laboratory, School of Chinese Medicine, Jinan University, Guangzhou, China, between December 2019 and February 2020. The study was approved by the Ethical Committee of our institute (Date: July 3, 2020). A three-dimensional finite element model of L3-S1 was built and well validated in one of our previously published studies^11^ (Fig.1). The model consisted of vertebral bodies, endplates, intervertebral discs, facet joints, and spinal ligaments. The intervertebral disc was composed of annulus fibrosus and nucleus pulposus, in which the matrix was modeled based on the incompressible, hyperelastic Mooney-Rivlin formulation, and c1 and c2 were defined according to previously published studies.^12^ The contact between facet joints was surface-to-surface contact with a friction coefficient of 0.1. Ligaments included anterior longitudinal ligament, ligamentum flavum, posterior longitudinal ligament, intertransverse ligament, interspinous ligament, supraspinous ligament, and capsular ligament.

The PLIF model was developed based on the healthy model, in which the supraspinous ligament, interspinous ligament, ligamentum flavum, and posterior longitudinal ligament as well as the inferior portion of lamina and medial half of the facet joints of L4 were removed. Two polyether-ether-ketone (PEEK) cages were inserted into the disc space after intervertebral disc was resected (Fig.1). The contact between vertebral body and cage was defined as “bonded” to simulate solid fusion. The spinal fixation including four pedicle screws (6.0 mm diameter) and two rods (5.5 mm diameter) was made of titanium alloy. The PLIF with osteoporosis model was built based on the PLIF model by adjusting material properties. All the material properties used in this study were defined based on previously published literature (Table-I and II).^11^-^14^ The inferior surface of the sacrum in each model was entirely fixed under all loading conditions. A preload of 400 N combined with a moment of 7.5 Nm in direction of flexion, extension, axial rotation, and lateral bending was applied on the superior surface of L3 in the healthy model. For the PLIF and PLIF with osteoporosis models, based on a hybrid test method,^15^ a preload of 400N combined with an adjusted moment was imposed on the superior surface of L3 to obtain the same total ROM (L3-S1) as the healthy model in each direction. The intradiscal pressure, shear stress on annulus fibrosus, and ROM at L3-L4 and L5-S1 were calculated and compared.

**Table-I:** Material properties used in finite element models of the lumbar spine (Part-1).

*Healthy model and PLIF model*			*PLIF with osteoporosis model*			

*Structures*	*Young’s modulus (MPa)*	*Poisson’s ratio*	*Element type*	*Young’s modulus (MPa)*	*Poisson’s ratio*	*Element type*
Cortical bone	Exx=11300	Vxy=0.484		Exx=7571	Vxy=0.484	
	Eyy=11300	Vyz=0.203	Hex	Eyy=7571	Vyz=0.203	Hex
	Ezz=22000	Vxz=0.203		Ezz=14740	Vxz=0.203	
	Gxy=3800			Gxy=2546		
	Gyz=5400			Gyz=3618		
	Gxz=5400			Gxz=3618		
Cancellous bone	Exx=140	Vxy=0.45		Exx=47.6	Vxy=0.45	
	Eyy=140	Vyz=0.315	Tetra	Eyy=47.6	Vyz=0.315	Tetra
	Ezz=200	Vxz=0.315		Ezz=100	Vxz=0.315	
	Gxy=48.3			Gxy=16.42		
	Gyz=48.3			Gyz=24.15		
	Gxz=48.3			Gxz=24.15		

**Table-II:** Material properties used in finite element model of the lumbar spine (Part-2).

*Structures*	*Young’s modulus (MPa)*	*Poisson’s ratio*	*Element type*
Endplate	500	0.25	Hex
Posterior structure	3500	0.30	Tetra
Sacrum	5000	0.20	Tetra
Facet joint	3500	0.25	Hex
Nucleus	Mooney-Rivlin C1 = 0.12, C2 = 0.03		Hex
Annulus	Mooney-Rivlin C1 = 0.18, C2 = 0.045		Hex
Polyether ether ketone Cage	3600	0.25	Tetra
Spinal fixation	110000	0.3	Tetra
Annulus fiber layers	360 - 550	0.3	Spring
Ligament	Calibrated deflection-force curves		Spring

## RESULTS

In this study, under 7.5 Nm moment and 400 N preload, the ROM at L3–S1 segment in the healthy model was 12.2°, 15.6°, 13°, and 7.7° in flexion, extension, lateral bending, and axial rotation, respectively. The identified moment which caused the same ROM was 12.5Nm, 12.3Nm, 11.5Nm, and 11Nm for PLIF model, and 10.8Nm, 11.3Nm, 10.4Nm, and 10.7Nm for PLIF with osteoporosis model in flexion, extension, lateral bending, and axial rotation, respectively.

**Fig.2:**
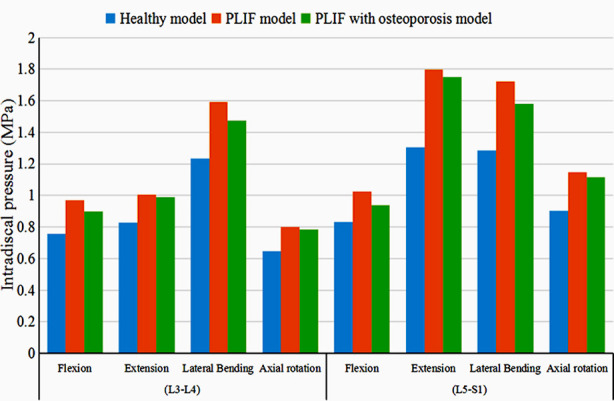
The distribution of intradiscal pressure.

**Fig.3:**
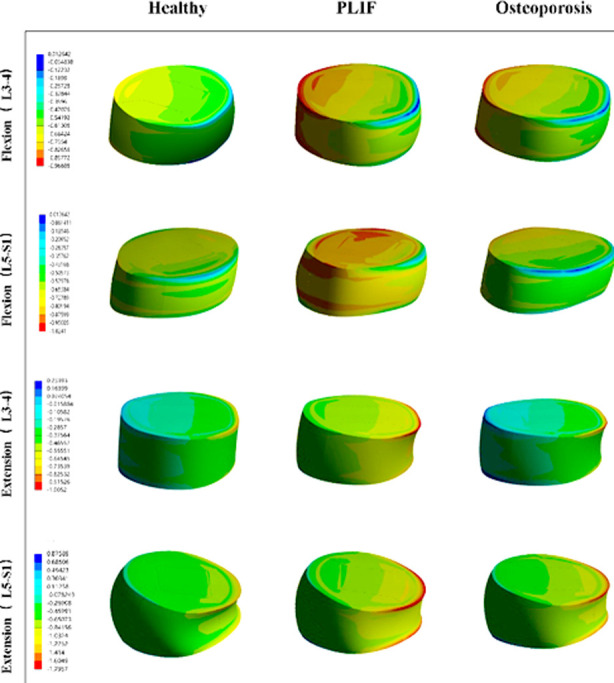
The intradiscal pressure nephogram of the models in flexion and extension.

The intradiscal pressure at L3-L4 and L5-S1 is shown in Fig. 2 and 3. In each direction, the highest value at L3-L4 and L5-S1 levels was found in the PLIF model, and the lowest value in the healthy model. Compared with the PLIF with osteoporosis model, the intradiscal pressure at L3-L4 level in PLIF model increased by 7.61%, 1.66%, 8.05%, and 2.34%, and the value at L5-S1 level increased by 9.27%, 2.55%, 8.92%, and 2.77% in flexion, extension, lateral bending, and axial rotation, respectively (Fig. 2 and 3).

**Fig.4:**
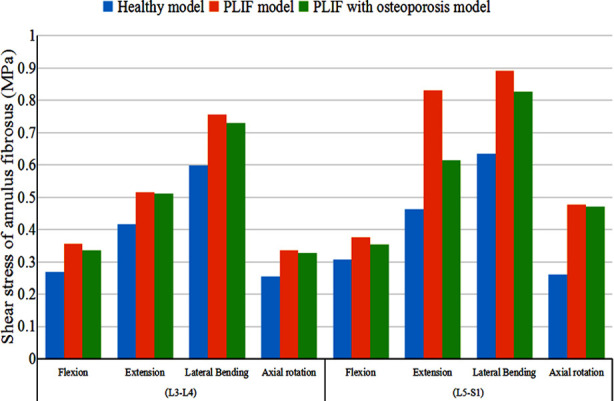
The distribution of shear stress on annulus fibrosus.

The shear stress on annulus fibrosus at L3-4 and L5-S1 levels is shown in Fig.4. In each direction, the highest value at L3-4 and L5-S1 was found in the PLIF model, and the lowest value in the healthy model. Compared with the PLIF with osteoporosis model, the shear stress at L3-4 level in PLIF model increased by 6.32%, 0.86%, 3.74%, and 2.60%, and the value at L5-S1 level increased by 6.50%, 35.06%, 7.81%, and 1.50% in flexion, extension, lateral bending, and axial rotation, respectively (Fig.4).

**Fig.5:**
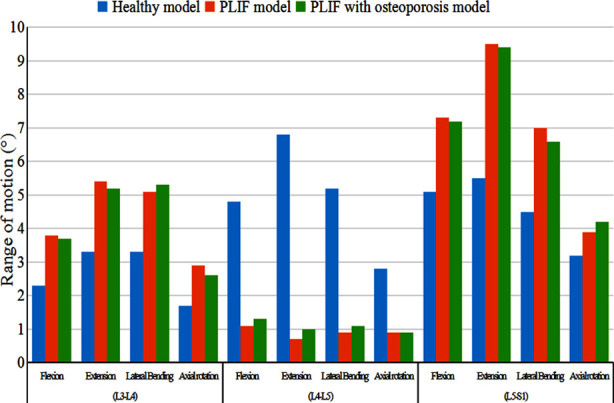
The distribution of range of motion.

The ROM at L3-L4, L4-L5, and L5-S1 levels is shown in Figure 5. At L4-L5, the largest value in each direction was found in the healthy model, and the smallest value in the PLIF model. At L3-L4 and L5-S1, the highest value in most directions was found in the PLIF model, followed by PLIF with osteoporosis model, and the lowest value was found in the healthy model. However, the largest value at L3-L4 level in lateral bending and at L5-S1 level in axial rotation was found in PLIF with osteoporosis model (Fig.5).

## DISCUSSION

In the current study, we evaluated the biomechanical effects of osteoporosis on adjacent segments after PLIF. To the best of our knowledge, few studies have been performed on the issue, the study may help surgeons better understand the correlation between ASD and osteoporosis. Many studies have confirmed that increased intradiscal pressure, shear stress on annulus fibrosus, and ROM of the lumbar spine are closely associated with ASD.^8^,^16^-^18^ Subsequently, in this study, we investigated the influence of osteoporosis on ASD using these outcomes. In addition, as the hybrid method can provide a comprehensive and appropriate evaluation of ASD after lumbar fusion, we used it for calculation.^15^,^19^

We found the values of the above-mentioned outcomes at L3-4 and L5-S1 levels were larger in the PLIF and the PLIF with osteoporosis models than the healthy model, demonstrating that PLIF can accelerate the development of ASD. In addition, each outcome was larger in the PLIF with osteoporosis model than the healthy model, indicating PLIF with osteoporosis also influence the progression of ASD adversely. Some studies have advocated that increased stiffness and stress concentration after lumbar fusion result in biomechanical changes and the occurrence of ASD at the adjacent levels.^12^,^20^ In the current study, although the Young’s modulus of cortical and cancellous bones was significantly decreased, the stiffness of L3-S1 segment was still higher in the PLIF with osteoporosis model than the healthy model, so it resulted in higher values of the outcomes at the adjacent levels.

In addition, compared with the PLIF with osteoporosis model, the PLIF model presented with higher values of intradiscal pressure and shear stress in each direction, demonstrating more significant stress concentration at its adjacent levels. In terms of ROM, as the two PLIF models have the same geometry, so their ROM distributions were similar. However, in flexion, extension and lateral bending the ROM at L4-L5 level was larger in the PLIF with osteoporosis model than the PLIF model, and in axial rotation the value was equal to the PLIF model. Subsequently, in most directions the outcomes were larger in the PLIF model than the PLIF with osteoporosis model.

From the angle of biomechanics, a functional spinal unit can alter in loading transfer because of osteoporosis, which leads to changed compression stiffness and strain distributions as well as magnitudes.^21^,^22^ In this study, the two PLIF models presented with the same total ROM, but the moment imposed on the PLIF with osteoporosis model was smaller, indicating a decrease of stiffness in this model. As the unique difference was material properties between the two models, we believe that it is osteoporosis which leads to a decreased intradiscal pressure, shear stress, and ROM at the adjacent levels in the PLIF with osteoporosis model. Subsequently, we conclude that osteoporosis can mitigate the influence of PLIF on adjacent segments.

### Limitations of the study

First of all, the models used in this study were reconstructed based on the lumbar spine of a healthy young man. In clinical practice patients undergoing PLIF usually have pathological changes, such as osteophyte formation,^23^ spinal stenosis and spondylolisthesis,^24^ which may also interfere the development of ASD, but were not considered in this study. Secondly, the muscles around the spine were ignored to simplify the calculation, while the loading conditions used in the current study may not be completely representative of the real physiologic situation. Thirdly, the current issue may be related to many fields of research, such as molecular biology, cell biology, biomechanics, and immunohistochemistry. while in this study we only analyzed the influence of osteoporosis on ASD from the angle of biomechanics. Hence, more studies need to be performed in the future.

### Author`s Contribution:

**SJT** conceived and designed the project, and are responsible and accountable for the accuracy and integrity of the work.

**CCZ, MMC, and RWZ** did model creation, calculation and manuscript writing,

**SJT and CCZ** did review and final approval of manuscript.

## References

[1] Ke W, Wang B, Hua W, Lu S, Li X, Yang C (2020). Biomechanical Evaluation of the Sacral Slope on the Adjacent Segment in Transforaminal Lumbar Interbody Fusion:A Finite Element Analysis. World Neurosurg.

[2] Bagheri SR, Alimohammadi E, Zamani Froushani A, Abdi A (2019). Adjacent segment disease after posterior lumbar instrumentation surgery for degenerative disease:Incidence and risk factors. J Orthop Surg (Hong Kong).

[3] Li J, Xu W, Zhang X, Xi Z, Xie L (2019). Biomechanical role of osteoporosis affects the incidence of adjacent segment disease after percutaneous transforaminal endoscopic discectomy. J Orthop Surg Res.

[4] Yousefzadeh N, Kashfi K, Jeddi S, Ghasemi A (2020). Ovariectomized rat model of osteoporosis:a practical guide. Excli J.

[5] Zhou Z, Tian FM, Gou Y, Wang P, Zhang H, Song HP (2016). Enhancement of Lumbar Fusion and Alleviation of Adjacent Segment Disc Degeneration by Intermittent PTH(1-34) in Ovariectomized Rats. J Bone Miner Res.

[6] Hashimoto K, Aizawa T, Kanno H, Itoi E (2019). Adjacent segment degeneration after fusion spinal surgery-a systematic review. Int Orthop.

[7] Min JH, Jang JS, Jung B, Lee HY, Choi WC, Shim CS (2008). The clinical characteristics and risk factors for the adjacent segment degeneration in instrumented lumbar fusion. J Spinal Disord Tech.

[8] Park P, Garton HJ, Gala VC, Hoff JT, McGillicuddy JE (2004). Adjacent segment disease after lumbar or lumbosacral fusion:review of the literature. Spine (Phila Pa 1976).

[9] Tang S (2015). Comparison of posterior versus transforaminal lumbar interbody fusion using finite element analysis. Influence on adjacent segmental degeneration. Saudi Med J.

[10] Lin GX, Park CK, Hur JW, Kim JS (2019). Time Course Observation of Outcomes between Minimally Invasive Transforaminal Lumbar Interbody Fusion and Posterior Lumbar Interbody Fusion. Neurol Med Chir (Tokyo).

[11] Zhang R Finite element analysis of two types of oblique pulling manipulations for lumbar disc herniation based on different degrees of degenerative models with nerve structures[D].

[12] Tang S, Rebholz BJ (2013). Does lumbar microdiscectomy affect adjacent segmental disc degeneration?A finite element study. J Surg Res.

[13] Su X, Shen H, Shi W, Yang H, Lv F, Lin J (2017). Dynamic characteristics of osteoporotic lumbar spine under vertical vibration after cement augmentation. Am J Transl Res.

[14] Huang YP, Du CF, Cheng CK, Zhong ZC, Chen XW, Wu G (2016). Preserving Posterior Complex Can Prevent Adjacent Segment Disease following Posterior Lumbar Interbody Fusion Surgeries:A Finite Element Analysis. PLoS One.

[15] Erbulut DU, Zafarparandeh I, Hassan CR, Lazoglu I, Ozer AF (2015). Determination of the biomechanical effect of an interspinous process device on implanted and adjacent lumbar spinal segments using a hybrid testing protocol:a finite-element study. J Neurosurg Spine.

[16] Jiang S, Li W (2019). Biomechanical study of proximal adjacent segment degeneration after posterior lumbar interbody fusion and fixation:a finite element analysis. J Orthop Surg Res.

[17] Tang S, Meng X (2011). Does disc space height of fused segment affect adjacent degeneration in ALIF?A finite element study. Turk Neurosurg.

[18] Kim HJ, Chun HJ, Moon SH, Kang KT, Kim HS, Park JO (2010). Analysis of biomechanical changes after removal of instrumentation in lumbar arthrodesis by finite element analysis. Med Biol Eng Comput.

[19] Panjabi MM (2007). Hybrid multidirectional test method to evaluate spinal adjacent-level effects. Clin Biomech (Bristol Avon).

[20] Tang S, Rebholz BJ (2011). Does anterior lumbar interbody fusion promote adjacent degeneration in degenerative disc disease?A finite element study. J Orthop Sci.

[21] Xiao ZF, Su GY, Hou Y, Chen SD, Zhao BD, He JB (2020). Mechanics and Biology Interact in Intervertebral Disc Degeneration:A Novel Composite Mouse Model. Calcif Tissue Int.

[22] Li J, Xu W, Zhang X, Xi Z, Xie L (2019). Biomechanical role of osteoporosis affects the incidence of adjacent segment disease after percutaneous transforaminal endoscopic discectomy J Orthop Surg Res.

[23] Watanabe K, Yamazaki A, Morita O, Sano A, Katsumi K, Ohashi M (2011). Clinical outcomes of posterior lumbar interbody fusion for lumbar foraminal stenosis:preoperative diagnosis and surgical strategy. J Spinal Disord Tech.

[24] Okuda S, Nagamoto Y, Matsumoto T, Sugiura T, Takahashi Y, Iwasaki M (2018). Adjacent segment disease after single segment posterior lumbar interbody fusion for degenerative spondylolisthesis:minimum 10 years follow-up. Spine (Phila Pa 1976).

